# Supplementary material for: A Global Screen for Assembly State Changes of the Mitotic Proteome by SEC-SWATH-MS
Source: Cell Syst. 2020 Feb 26;10(2):133–155.e6. doi: 10.1016/j.cels.2020.01.001 (PMC7042714; doi:10.1016/j.cels.2020.01.001)

Q14BN4 | SLMAP_HUMAN | SLMAP KIAA1601 SLAP UNQ1847/PRO3577
Monomer MW [kDa]: 95.198 Monomer expected elution fraction: 41

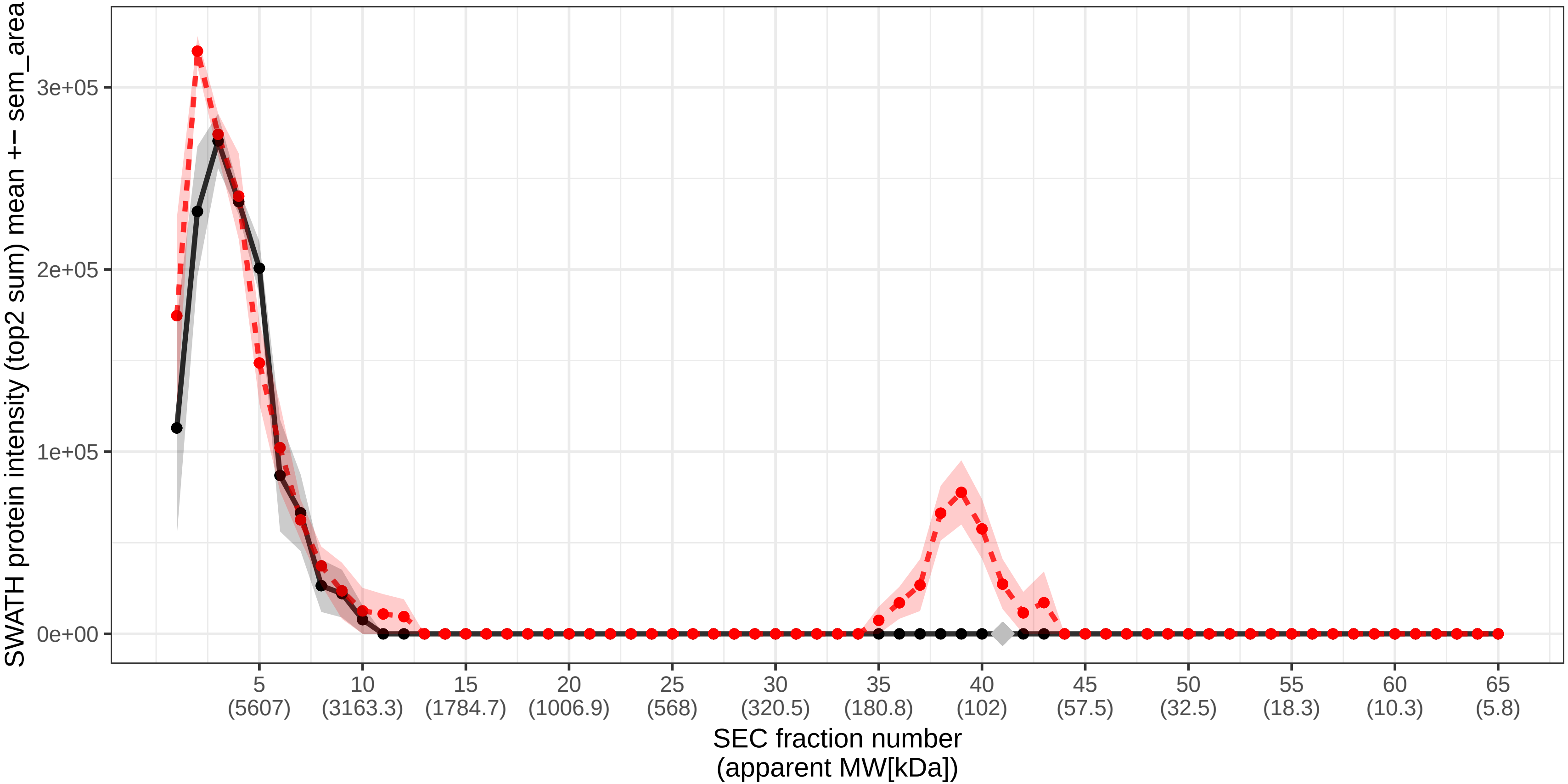

Supplement: Data S1. SEC-SWATH-MS Protein Chromatograms, Related to Figure 1 [file mmc6.zip › SECchrom_Q14BN4_SLMAP_HUMAN_SLMAP_KIAA1601_SLAP_UNQ1847_PRO.pdf]
